# The relationship between DNA methylation and *Reprimo* gene expression in gastric cancer cells

**DOI:** 10.18632/oncotarget.21296

**Published:** 2017-09-28

**Authors:** Junzhong Lai, Hanze Wang, Qianping Luo, Shanlu Huang, Shujin Lin, Yansong Zheng, Qi Chen

**Affiliations:** ^1^ Fujian Key Laboratory of Innate Immune Biology, Biomedical Research Center of South China, College of Life Science, Fujian Normal University Qishan Campus, Fuzhou, Fujian Province, China; ^2^ The First Affiliated Hospital of Fujian Medical University, Fuzhou, Fujian Province, China

**Keywords:** gastric cancer, reprimo, dna methylation, dna methyltransferase, zebularine

## Abstract

Reprimo (RPRM) is a tumor suppressor involved in the development of a number of malignant tumors including gastric cancer which is highly related to its gene hypermethylation. However, the regulation of RPRM gene expression by DNA methylation in gastric cancer is not well understood. We examined the RPRM gene methylation in gastric cancer tissues or plasma samples by bisulfite sequencing, and investigated the relationship between DNA methylation and the RPRM gene expression by quantitative reverse transcription-PCR and Western blotting. We found that the RPRM gene promoter region is hypermethylated in gastric cancer tissues (75%, 45/60), plasma samples (86.3%, 44/51) and various cancer cell lines (75%, 3/4), which is correlated with the decrease of RPRM gene expression. The hypermethylation-induced RPRM reduction can be recovered by treating with zebularine, a demethylating agent, and by inhibition of the DNA methyltransferases via RNA interference and CRISPR/Cas9-mediated gene knockout. In addition, we generated RPRM gene-knockout cells and studied the effects of the RPRM deficiency on tumor formation by inoculating these cells in mice. The data show that the loss of RPRM can promote tumorigenesis. These data suggest that the RPRM expression is inhibited by DNA methyltransferases and the RPRM normal function can be restored by treating with DNA methylation inhibitors. The study provides important information regarding the role of RPRM and its methylation related to gastric cancer development.

## INTRODUCTION

Gastric cancer is one of the leading causes of cancer-related death worldwide, and is highly prevalent in Asia, particularly in China [1, 2]. Despite recent progress in surgery and chemotherapy, the prognosis for gastric cancer is still not favorable. Therefore, early detection, better understanding of the molecular mechanisms and finding the new therapeutic strategies for this disease are imperative challenges [3]. Reprimo (RPRM), TP53 dependent G2 arrest mediator candidate, is a highly glycosylated protein and initially discovered as a putative tumor-suppressor involved in the regulation of p53-dependent G2 arrest of the cell cycle [4, 5]. Aberrant methylation of *RPRM* gene is shown to be closely related to the occurrence and development of gastric cancer [4].

DNA methylation plays a critical role in gene regulation, cellular differentiation and embryonic development [6]. Aberrant DNA methylation can disrupt normal gene functions and lead to various disease pathogenesis [7]. In carcinogenesis, aberrant DNA methylation appears in various ways including hypermethylation of tumor suppressor genes, aberrant expression of DNA methyltransferases (DNMTs), as well as hypermethylation of selected genes and repetitive sequences [8]. To reactivate the tumor suppressor genes by demethylation is an attractive therapeutic strategy of epigenetic therapy in order to rehabilitate aberrant cells [9].

DNMTs are the key regulators of DNA methylation and have crucial roles in epigenetic modification [10, 11]. They transfer methyl groups from S-adenosyl methionine to the 5’ unmethylated DNA cytosine ring to form 5-methylcytosine [12]. Three subtypes of DNMTs, including DNMT1, DNMT3A and DNMT3B, have been shown to have this activity, while the function of DNMT2 is currently unclear. DNMT1 is involved in maintaining DNA methylation by methylating newly synthesized strands of DNA during DNA replication [13, 14], whereas DNMT3A and DNMT3B are mainly involved in *de novo* methylation [15–20]. DNMT1 and DNMT3B have found to be overexpressed in gastric cancer [21].

DNA methyltransferase inhibitors 5-azacytidine and 5-aza-2-deoxycytidine have been developed, and used for treating myelodysplastic syndrome and acute myelogenous leukemia due to their abilities to reverse tumor suppressor gene methylation and restore gene expression [8, 22, 23]. These inhibitors function as nucleoside analogues and inhibit DNMT activity through covalent binding to the DNMT enzymes [4, 6]. The use of these two drugs has been limited due to their toxicity, instability, and low efficacy. Zebularine (1-(b-D-ribofuranosyl)-1, 2-dihydropyrimidin-2-one) is a cell-permeable chemically stable cytidine analog that was initially discovered as a deaminase inhibitor [24–26]. Zebularine is thought to be a better DNA methyltransferase inhibitor due to its relative low toxicity, high stability, and oral bioavailability [24, 27–37].

In the present study, we examined the DNA methylation of the *RPRM* gene promoter region derived from the cancer tissues and blood samples of gastric cancer, evaluated the relationship between RPRM promoter methylation and its gene expression in several cancer cell lines. The role of DNMTs in regulating RPRM methylation and expression was also studied by treating with zebularine, and by RNA interference and CRISPR/Cas9-mediated gene knockout. The implications of these results for the potential application of RPRM as a biomarker and DNMT inhibition-based therapeutics are discussed.

## RESULTS

### Cancer-related *RPRM* promoter methylation in gastric cancer

**Figure 1:**
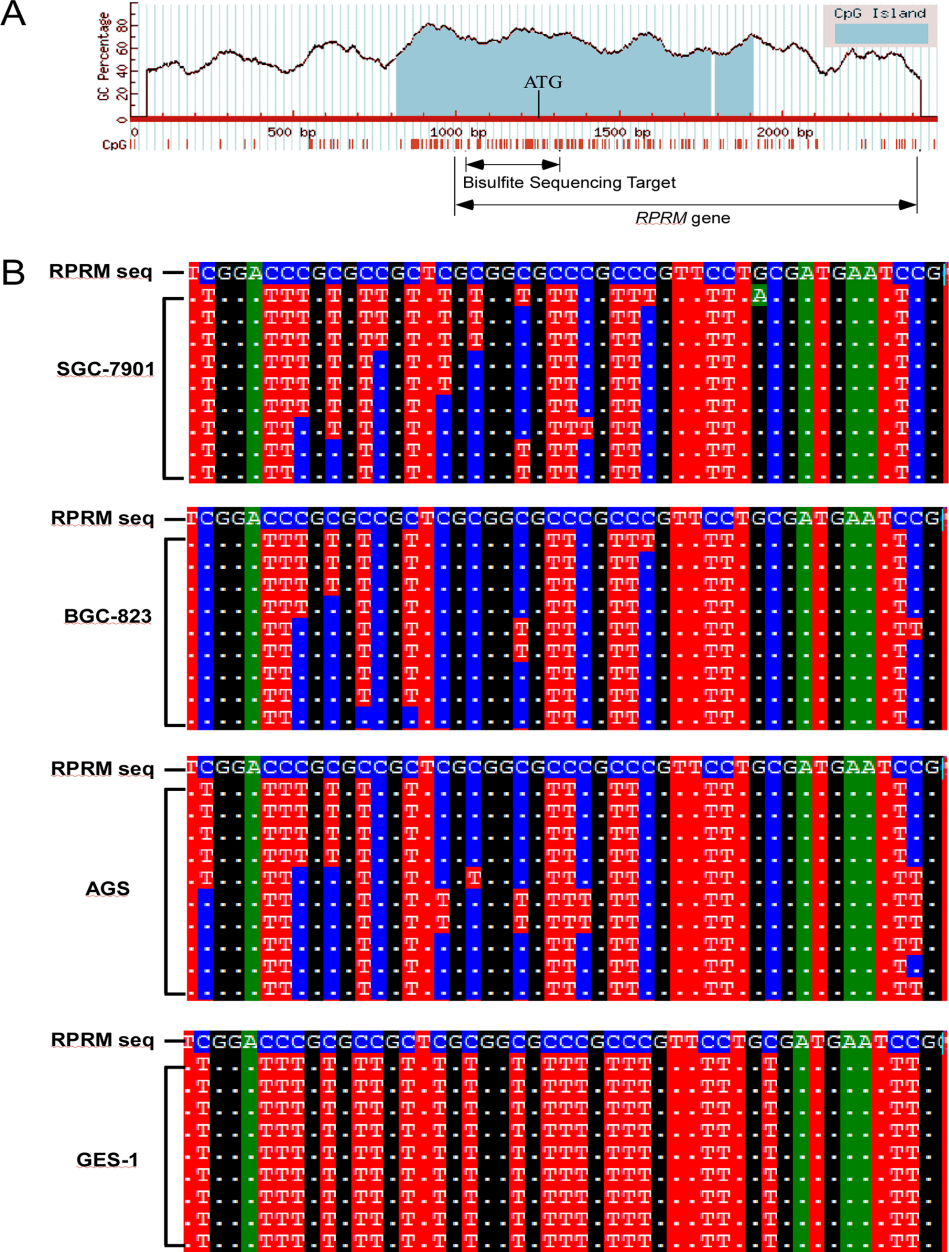
The methylation of *RPRM* promoter region analyzed by bisufite sequencing (**A**) The CpG islands of *RPRM* gene and its 5’-flanking region, predicted by MethPrimer. The RPRM gene, translation initiation sites (ATG), and the bisulfite primers target regions are indicated. (**B**) *RPRM* gene promoter bisulfite sequencing results of SGC-7901, BGC-823, AGS and GES-1 cell lines. Only a part of the sequencing results are presented for representation. The *RPRM* gene reference sequence (RPRM seq) is placed at top of each panel and bisulfite sequencing results of each clones from different cell lines are placed underneath, with unconverted (methylated) cytosine highlighted in blue color.

The location of CpG islands in the 5’-flanking region of *RPRM* gene was predicted by MethPrimer [4] and we picked a pair of bisulfite sequencing primers which span the 261 bp region containing 30 CpG sites (Figure 1). The methylation profiles of various tissue samples from 60 gastric cancer patients were studied by bisulfite sequencing and methylation-sensitive melt curve analysis (MS–MCA). The methylation in the *RPRM* promoter region occurred in 75.0% (45/60) of the primary human gastric cancer tissues, but only in 43.5% (20/46) of the corresponding adjacent normal tissues. A high occurrence of methylation (86.3%, 44/51) was also found in the plasma samples of the gastric cancer patients, but rarely found in plasma (7.9%, 3.38) or in PBMC samples (2.4%, 1/49) of healthy adults (Table 1). The *RPRM* methylation was also found in all of the 2 gastric cancer cell lines, BGC-823 and AGS, but not in the gastric epithelium-immortalized cell line GES-1 (Figure 1).

**Table 1:** Methylation of different sample types

Sample Types	Gastric Cancer			All	Healthy	
	Ca. Tissues	Para. Tissues	Plasma	PBMC		Plasma
Samples	60	46	51	49		38
Methylated	45	20	44	1		3
Met%	75.0%	43.5%	86.3%	2.04%		7.9%

### Transcriptional silencing by *RPRM* promoter methylation

**Figure 2:**
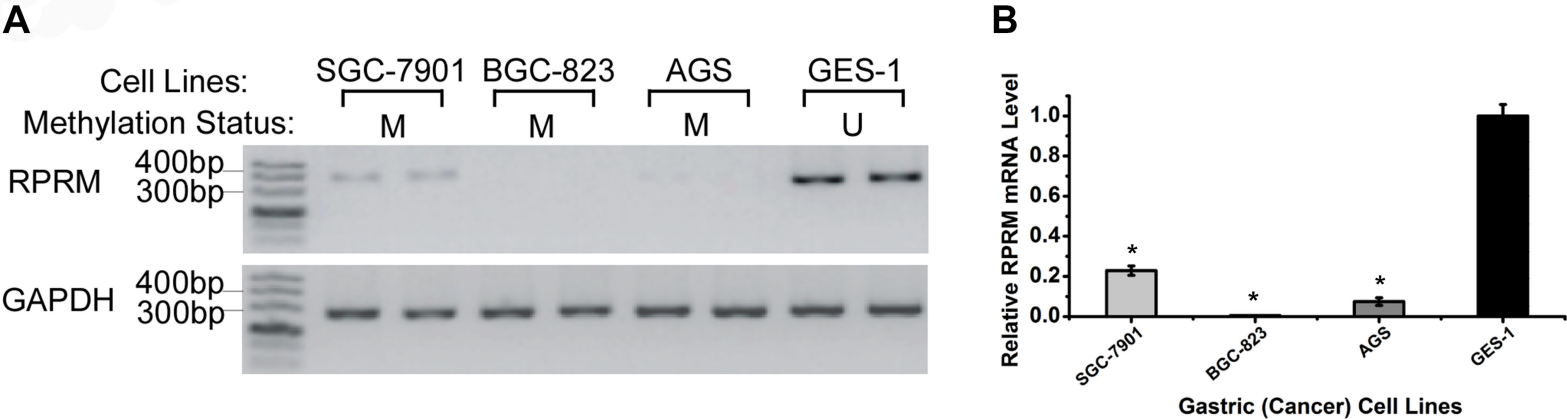
The RPRM mRNA expression in four cell lines (**A**) The mRNA transcription levels of RPRM in SGC-7901, BGC-823, AGS and GES-1 cell lines analyzed by RT-PCR. M, refers to methylated status of the RPRM promoter; U, refers to unmethylated status of the RPRM promoter. The levels of RPRM mRNAs in SGC-7901, BGC-823, AGS cell lines are low relative to that in the GES-1 cell line. (**B**) The statistical presentation of A. The mRNA transcription level is analyzed by densitometry using NIH Image J software and the fold changes of the mRNA expression is normalized to the GAPDH control. The data are analyzed by Student’s-t test and presented as mean ± SD (*n* = 3, ^*^p < 0.05).

To better understand the mechanism involved in the *RPRM* expression, we studied the relationship between the *RPRM* promoter methylation and its expression. To ask if the RPRM methylation resulted in transcriptional silencing of *RPRM* in gastric cancer, the mRNA expression level was evaluated by RT-PCR in the above four cancercell lines. The *RPRM* methylation statuses of these samples were also indicated according to the bisulfite sequencing results (Figure 1). In anticorrelation with the *RPRM* promoter methylation, the *RPRM* mRNA expression was almost undetectable in two of four cancer cell lines. In contrast, we found weak *RPRM* mRNA expression in the SGC-7901 cell line and strong *RPRM* mRNA expression in the GES-1 cell line (Figure 2). Thus, the *RPRM* mRNA transcription was inversely correlated with the *RPRM* methylation.

### Zebularine restores the RPRM expression by inhibiting DNA methylation

**Figure 3:**
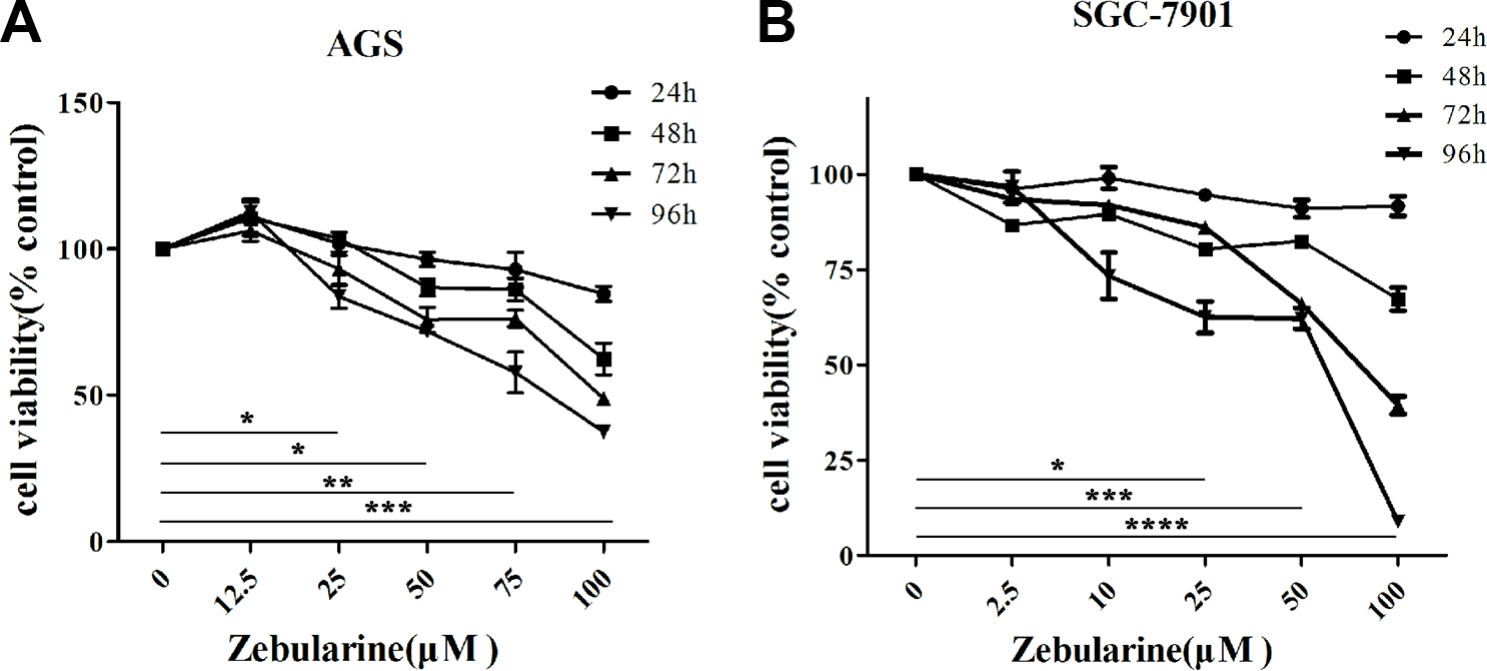
The effects on cell viability by zebularine in the designated doses and time points AGS (**A**) and SGC-7901 (**B**) cells were treated with different doses of zebularine for 24, 48, 72 and 96 h, and measured by the MTT assay, respectively. SGC-7901 cells appear to be more sensitive to zebularine treatments. The above experiments were repeated at least 6 times. The data of 96 hour treatments were analyzed by Student’s-t test and presented as mean changes ± SD (*n* = 6, ^*^p < 0.05, ^**^p < 0.01, ^***^p < 0.001, ^****^p < 0.0001).

Previous studies have shown that hypermethylation of the promoter region is one of the principal mechanisms to silence the *RPRM* expression [4, 38]. To investigate the effects of DNA methylation on gene expression, we selected a demethylating drug, zebularine, and examined its ability to affect the *RPRM* expression in the AGS and SGC-7901 cells. Its effects on cell viability were first assessed by MTT assays to determine the optimal concentrations of zebularine used for the following studies. For AGS cells, the cell viability was reduced more than 50% after 96 h treatment with 50 μM zebularine, while for SGC-7901 cells, the cell viability was reduced to 50% after 72 h treatment with 12.5 μM zebularine (Figure 3). Thus, AGS cells appeared to have a stronger tolerance relative to SGC-7901 cells.

**Figure 4:**
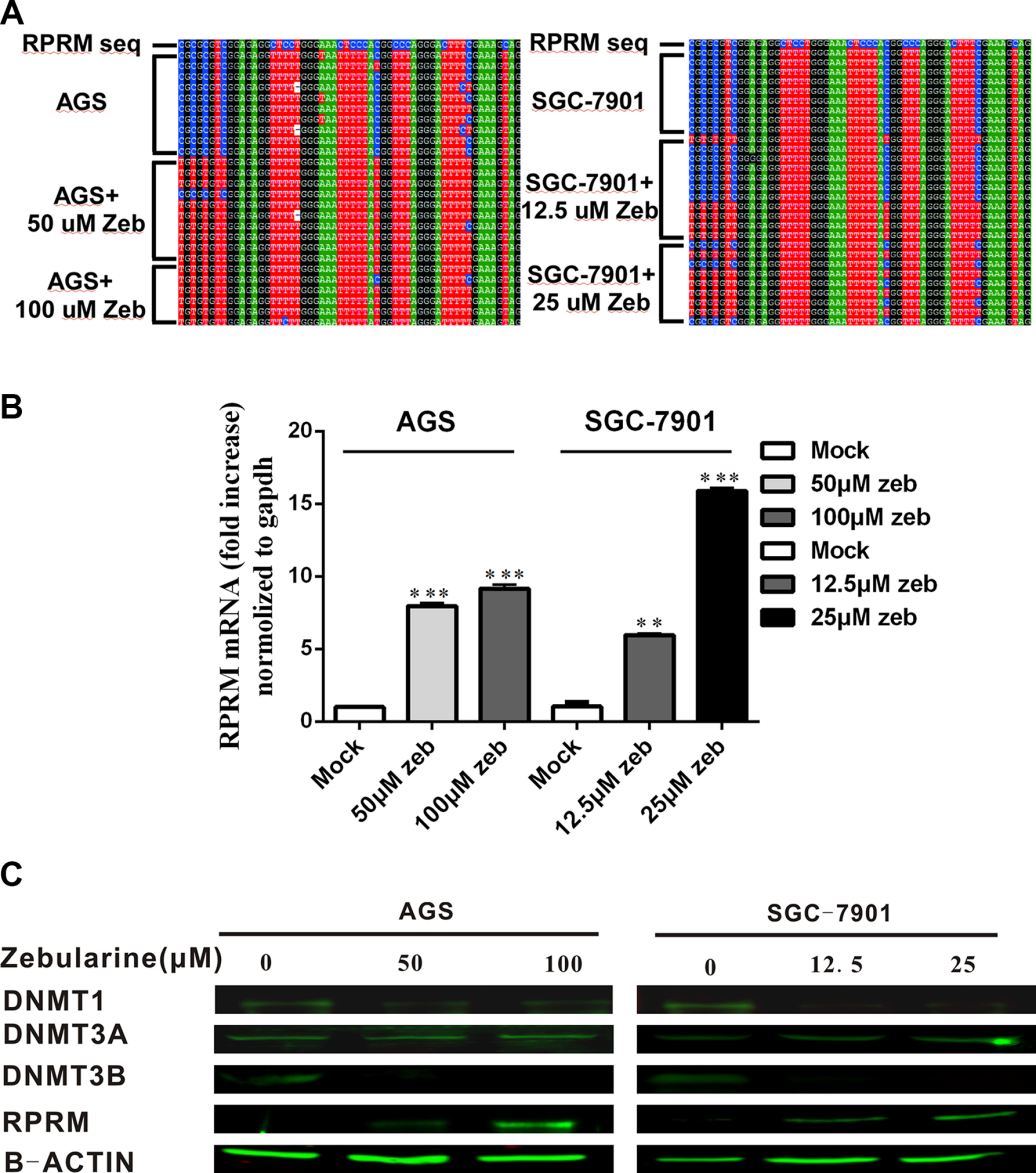
Demethylation effects of zebularine and its impact on the mRNA and protein expression of RPRM and DNMTs (**A**) The methylation of RPRM promoter region was analyzed by bisulfite sequencing in AGS cells treated with 50 μM and 100 μM zebularine for 96 h, respectively; and SGC-7901 cells treated with 12.5 μM and 25 μM zebularine for 96 h, respectively. The blue C denotes methylated CpG sites. (**B**) The mRNA transcription levels of DNMT1, DNMT3B and RPRM after treatment with zebularine at the designated doses, and analyzed by qRT-PCR in AGS and SGC-7901 cell lines, respectively. The fold changes of RPRM mRNA expression are normalized to the internal GAPDH control. The statistical analyses are performed by Student’s-t test and the data are presented as mean ± SD (*n* = 3, ^**^p < 0.01, ^***^p < 0.001). (**C**) The protein expression levels of DNMT1, DNMT3A, DNMT3B and RPRM in SGC-7901 and AGS cell lines after treatment with zebularine at the designated doses and analyzed by Western blotting.

Next, AGS and SGC-7901 cells were treated with the different concentrations of zebularine to test its demethylating effects on the *RPRM* gene. The bisulfite sequencing results showed that the percentage of the *RPRM* gene methylation was reduced after zebularine treatments in both AGS and SGC-7901 cells (Figure 4).

Simultaneously, zebularine’s effects on the *RPRM* gene expression at the transcription and translational levels in AGS and SGC-7901 cells were also assessed. As shown in Figure 4B and C, zebularine treatments led to an increase of RPRM expression in both AGS and SGC-7901 cells.

We also examined the effects of zebularine on the expression of DNMTs by RT-PCR and Western blotting. Interestingly, zebularine treatments caused a dose-dependent depletion of DNMT1 and DNMT3B proteins, but not DNMT3A in AGS and SGC-7901 cells (Figure 4C). We did not observe the zebularine effect on the mRNA expression of DNMTs (Data not shown). These data indicate that zebularine affects not only DNMT activities as previously reported [27, 39], but also the DNMT expression.

### Silencing DNMTS increased the expression of RPRM in two cancer cell lines

**Figure 5:**
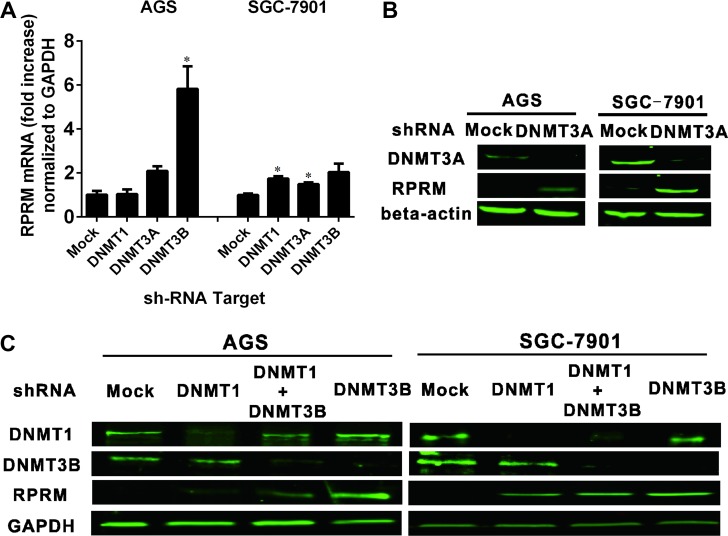
The relationship between DNMTs and RPRM expression evaluated by RNA interference and gene expression (**A**) The effects on the RPRM mRNA transcription by RNAi knockdown of DMNTs in AGS and SGC-7901 cells, respectively. The fold changes of the RPRM mRNA transcription were analyzed by qRT-PCR and normalized to the GAPDH control. The statistical analyses are performed by Student’s-t test and the data are presented as mean ± SD (*n* = 3, ^*^p < 0.05). (**B**) The protein expression levels of DNMT3A and RPRM in AGS and SGC-7901 cell lines after RNA interference of DNMT3A in AGS and SGC-7901, respectively, and analyzed by Western blotting. (**C**) The protein expression levels of DNMTs and RPRM after RNA interference of designated DNMTs in AGS and SGC-7901 cell lines, resepectively, and analyzed by Western blotting. The RPRM protein (12 kD) is detected at near 38 kD due to its heavy glycosylation[4]. The above qRT-PCR and Western blotting experiments were repeated at least three times.

To assess the roles of DNMTs in DNA methylation, we knocked down each of the DNMTs by RNA interference. As shown in Figure 5, the shRNAs significantly inhibited the expression of DNMT1, DNMT3A, and DNMT3B at the mRNA and protein levels. At the same time, knockdown of DNMT1, DNMT3A, DNMT3B, or both DNMT1 and DNMT3B significantly restored the RPRM expression in both AGS and SGC-7901 cells (Figure 5B and 5C).

**Figure 6:**
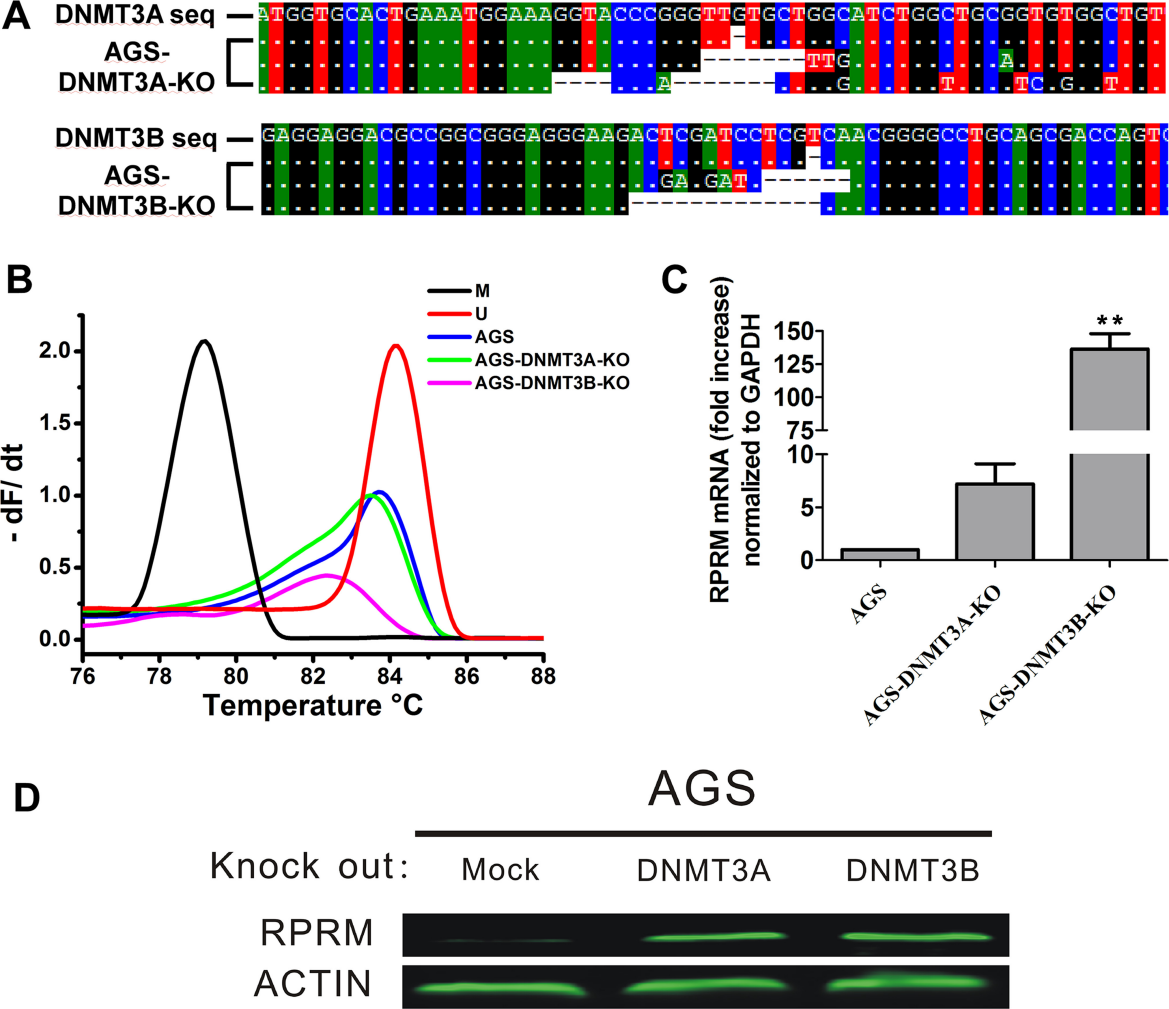
CRISPR/Cas9-mediated DNMT3A or DNMT3B knockout increases the RPRM expression (**A**) Verification of DNMT3A and DNMT3B-knockout monoclonal cells by sequencing. (**B**) The methylation of RPRM promoter region was analyzed by methylation-sensitive melt curve analysis (MS-MCA) as described previously. U indicates the unmethylated melt curve while M represents the melt curve of the full methylated control. Knockout of DNMT3a or DNMT3B causes the methylation-sensitive melt curve to move towards the unmethylated configuration. (**C**) The qRT-PCR data show that the mRNA transcription level of RPRM is increased by DNMT3A or DNMT3B knockout in the AGS cells. The changes of RPRM mRNA expression are normalized to the internal GAPDH control. The statistical analyses are performed by Student’s-t test and the data presented as mean ± SD (*n* = 3, ^**^p < 0.01). (**D**) The Western blotting data show that the RPRM level is increased by DNMT3A or DNMT3B knockout in the AGS cells.

To further validate the effects of DNMTs on the RPRM expression, we used the CRISPR/Cas9 technology to knock out DNMT1, DNMT3A and DNMT3B in AGS cells. We were not able to obtain DNMT1-knockout cell clones due to the extensive cell death induced by DNMT1 knockout. But several DNMT3A and DNMT3B-knockout cell clones were selected and confirmed by sequencing verification (Figure 6). Knocking out of DNMT3A or DNMT3B, especially the latter one, resulted in a significant increase of the RPRM mRNA by decreasing the RPRM promoter methylation as shown by qRT-PCR analyses (Figure 6). These data suggest an inverse correlation between DNMT functions and RPRM expression.

### RPRM is localized in the cytoplasm and its expression can be recovered by DNMT gene silencing

**Figure 7:**
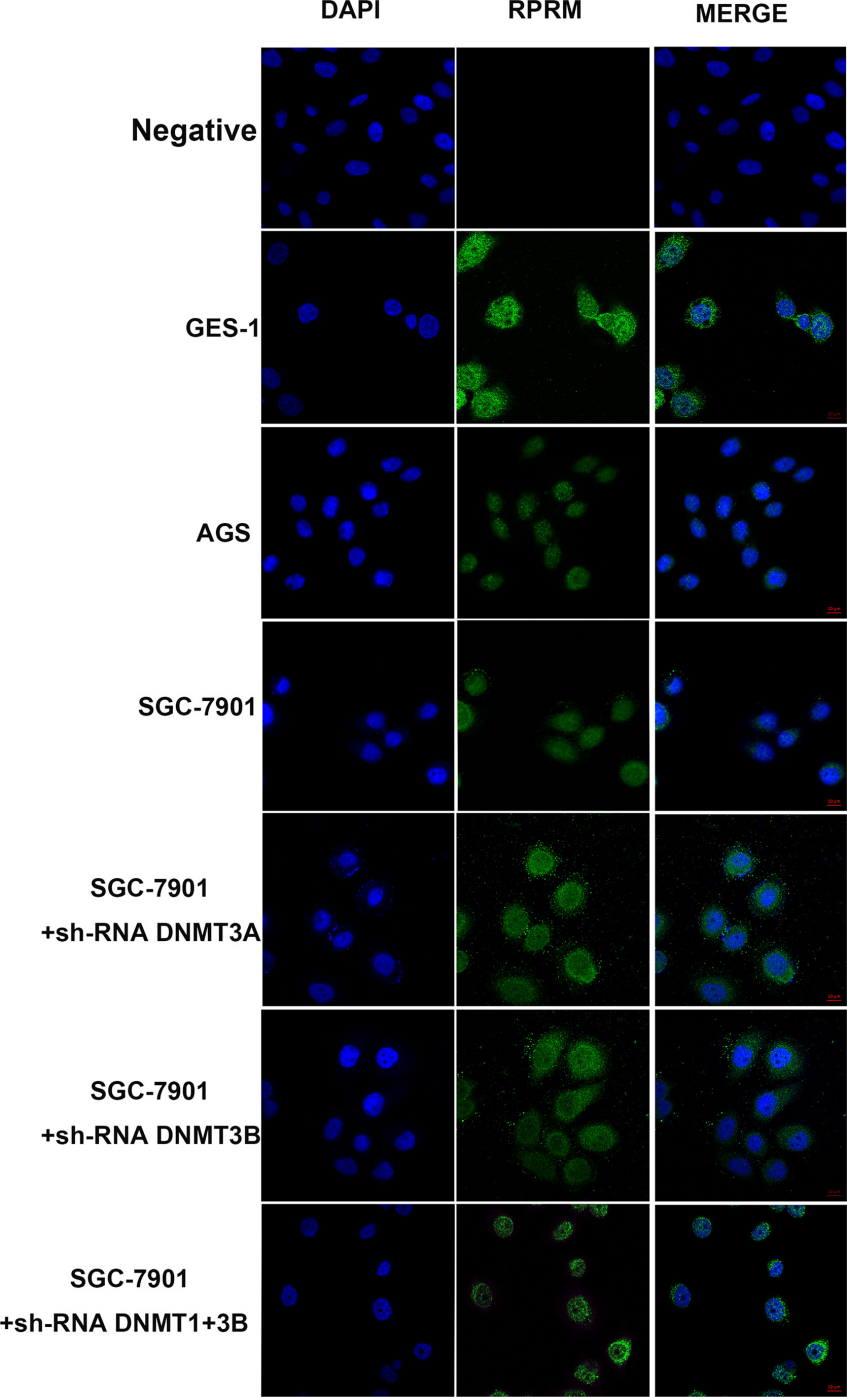
Immunofluorescence analyses of the expression and localization of RPRM in GES-1, AGS, SGC-7901, and SGC-7901 cells after RNAi knockdown of various DNMTs (**A**) RPRM is predominantly localized in the cytoplasm. Only faint or weak RPRM protein expression is seen in gastric cancer cell lines AGS and SGC-7901. However, the level of RPRM protein expression is significantly increased in GES-1 and SGC-7901 cells after silencing DNMT3A, DNMT3B and DNMT1+DNMT3B. The absence of primary antibody in the corresponding reaction was served as negative control. Images were taken by the inverted LSM 780/Axio Imager confocal microscope.

To further confirm the effects of DNMTs on the RPRM expression, we performed the immunofluorescent analyses by confocal microscopy in SGC-7901 cells transfected with the corresponding shRNA constructs and the empty vector, respectively. We observed faint or weak RPRM expression in the cytoplasm of AGS and SGC-7901 cells relative to GES-1 cells. However, the RPRM fluorescent signals were significantly brighter in the SGC-7901 cells transfected with the corresponding shRNA constructs by silencing DNMT3A, DNMT3B or both DNMT1 and DNMT3B (Figure 7), an outcome that is consistent with our Western blotting results.

### Loss of RPRM promoted the tumor formation.

**Figure 8:**
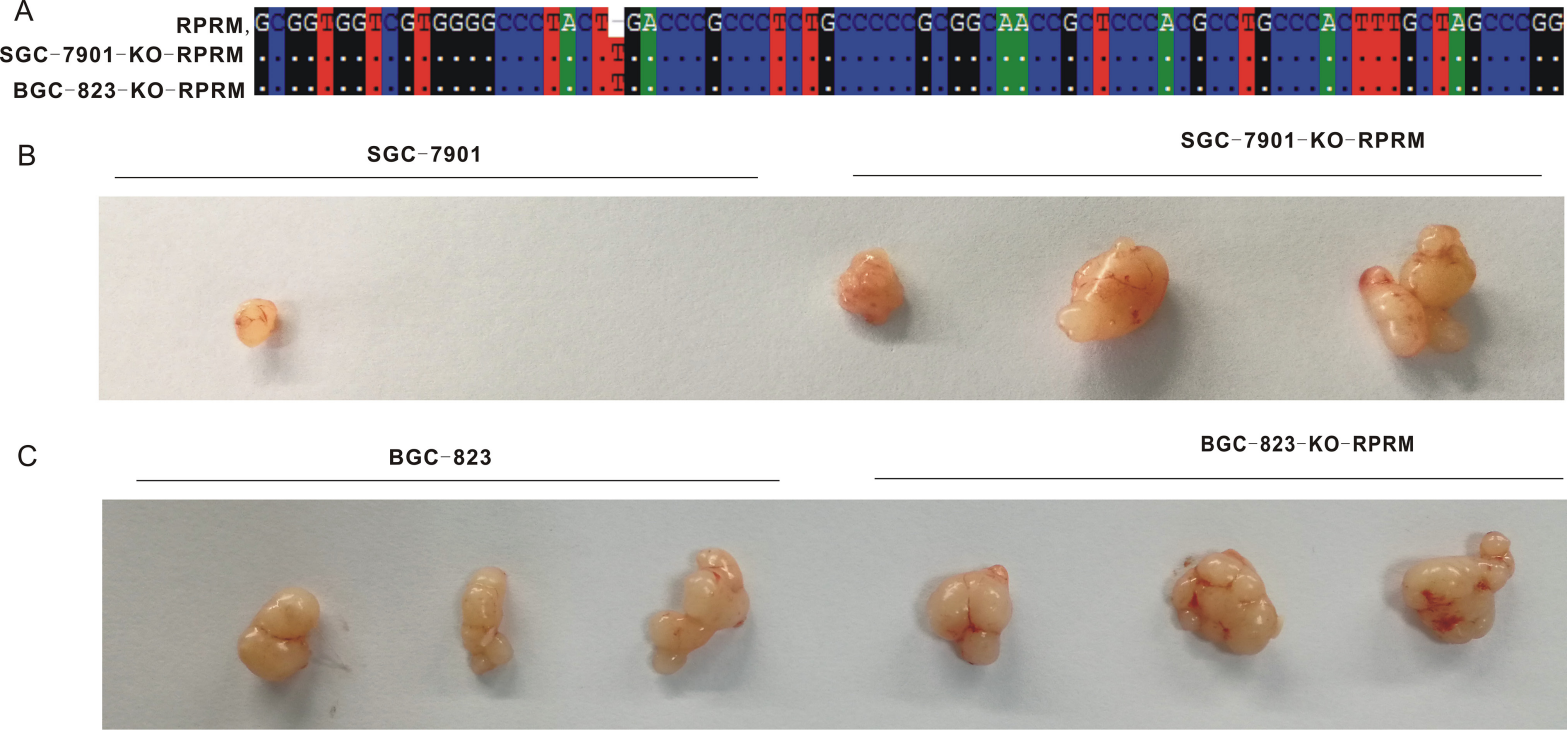
Loss of RPRM promoted tumorigenesis (**A**) Verification of SGC-7901 and BGC-823 Reprimo-knockout monoclonal cells by sequencing. (**B** and **C**) Tumors formed in nude mice after injected with SGC-7901 and BGC-823 and their reprimo gene knock out cell lines on the right shoulders (1 × 10^6^ cells each injection).

We next explored the role of RPRM in tumorigenesis in mice. We successfully generated the RPRM-deficient SGC-7901 and BGC-823 cell lines using the CRISPR/Cas9 technology (Figure 8A). The RPRM gene deficient SGC-7901 and BGC-823 cells were then subcutaneously inoculated onto the right shoulders of female BALB/cAJcl nude mice. The effect of the loss of RPRM on tumor formation was observed 10 days after inoculation. We found that 3 out of 5 mice displayed tumors arising from the RPRM-deficient SGC-7901 cells while 1 out of 5 mice had tumors arising from the control SGC-7901 cells (Figure 8B). Three out of 5 mice displayed tumors with both the RPRM-deficient and control BGC-823 cells (Figure 8C). However, the volume in all tumors inoculated with Reprimo-deficient cell lines were larger than those in the mice inoculated with counterpart control cells. These data suggest that the loss of RPRM can enhance tumor formation, which is consistent with its role functioning as a tumor suppressor.

## DISCUSSION

We used bisulfite sequencing and MS-MCA to evaluate the methylation status of the *RPRM* promoter in the tumor and para-cancerous tissues, and plasma samples of gastric cancer patients and normal healthy individuals. Consistent with our methylation-sensitive melt curve analyses [40], the *RPRM* promoter methylation was detected in all of the tumor tissues of gastric cancer patients. However, for some para-cancerous tissues, *RPRM* was also methylated, which suggests that the cellular fate of the para-cancerous tissues may have been changed or transformed, which is consistent with previous observations [4]. In agreement with the previous observations [41, 42], our data also show the *RPRM* hypermethylation in the plasma of gastric cancer patients in contrast to the plasma of control subjects. Thus, the aberrant *RPRM* promoter methylation may be manifested by using the gastric cancer patients’ plasma samples. The *RPRM* hypermethylation has been reported in premalignant conditions such as Barrett’s esophagus [43] and non-malignant gastric epithelia [1], as well as associated with cagA and vacA s1m1 alleles in the early gastric cancer progression. A role for RPRM in gastric cancer development has been postulated because the loss of RPRM expression was significantly associated with the progression from stage I to stages II-IV of gastric cancer. Therefore, the *RPRM* methylation may be a useful biomarker for early detection and prognosis of gastric cancer as suggested previously [4].

DNA hypermethylation of tumor suppressor genes is often associated with reduced expression or silencing of tumor suppressor genes. We also examined the correlation between the *Reprimo* methylation and its expression by RT-PCR in gastric cancer samples (Data not shown). We did not observe a consistent inverse correlation between the *Reprimo* methylation and its mRNA expression when compared the cancerous samples vs. their corresponding para-cancerous tissues, which is in line with previous report by Maturana et al. [44]. This result may be explained by the following reasons: 1) RT-PCR results might be compromised by the tissue samples, were highly mixed with various cell types including normal and tumorous cells; or 2) the more complex mechanism in addition to methylation may be involved in the regulation of mRNA expression. This question needs to be further addressed in the future. However, in cell lines, we did find that the decrease or loss of RPRM expression is accompanied by the RPRM promoter methylation, in line with previous findings [4, 38, 45]. The promoter methylation and decreased expression of RPRM in gastric cancer is consistent with the role of *RPRM* as a tumor suppressor gene. Therefore, to increase the RPRM expression and restore its function by demethylation therapy may also be considered for future cancer treatment.

In the present study, the *RPRM* methylation in cancer cell lines was reversed by zebularine, accompanied by partially restored RPRM expression. The DNMT1 and DNMT3B expression, but not DNMT3A expression, were also affected by zebularine in our studies (Figure 4C). However, we did not observe the zebularine effect on the mRNA expression of DNMTs (Data not shown). These data suggest that zebularine does not affect the transcription of DNMTs, but instead may affect the stability of DMNTs. We also found that SGC7901 cells are more sensitive to the zebularine treatment than AGS cells. Therefore, DNMTs are viable targets for DNMT inhibitors, although the sensitivities of these DNMTs may vary in the different cell types and zebularine may function differently in regards with different cancer types. In this context, caution is advised when using these inhibitors in treating different cancers and individuals.

The elevated expression of DNMTs is observed in a variety of malignancies, including gastric, lung, prostate, and colorectal tumors [46–55] and the inhibition of DNMT activity can strongly reduce the formation of tumors [56]. Inhibition of DNMTs correlates with reduction in tumorigenicity and increased expression of tumor suppressor genes [57]; Therefore it has been proposed as a good cancer treatment strategy [9]. To specifically target each of DNMTs, we used RNA interference and knockout strategies. We found that all three DNMTs including DNMT1, DNMT3A and DNMT3B were involved in the *RPRM* promoter methylation since silencing or knocking out any of above DNMTs was able to affect both methylation and expression of RPRM. These data also suggest that keeping the *RPRM* gene methylation requires the consonant functions among these DNMTs. These data may be used to explain why the percentage of methylation varied in the different tissue samples, but was not associated with the clinicopathological factors, including age and stage of tumor, as previously reported [4]. These data may also explain why no particular preferably methylated locus or region within the target sequence has been observed by us and others. But it remains unclear what causes the initiation of *RPRM* methylation.

RPRM has been suggested to be a tumor suppressor, but the direct evidence for this hypothesis is lacking. We showed here that the absence of RPRM was able to enhance tumor formation in mice. These data provided direct evidence regarding the role of RPRM in tumorigenesis and tumor suppression, which is supported by previous studies showing that overexpression of RPRM in gastric cancer cells reduces tumor formation and size [4]. We are currently generating the RPRM gene-null mutant mouse to further study the RPRM role in tumorigenesis.

In summary, we confirmed the hypermethylation of *RPRM* promoter in gastric cancer tissues and plasma. We further studied the relationship of the DNMT inhibitors on *RPRM* methylation and expression of DNMTs. We found that zebularine reduced the expression of *RPRM* mRNA and protein in SGC-7901 and AGS cell lines, accompanied by reduced expression of DNMT1 and DNMT3B. Direct inhibition of DNMTs by RNA interference and CRISPR/Cas9 knockout was also associated with reduction of the expression of RPRM mRNA and protein. The RPRM deficiency can promote tumor formation. These results have an important impact for better understanding the relationship between DNA methylation and RPRM expression, and its role in tumorigenesis.

## MATERIALS AND METHODS

### Clinical samples and cell culture

Tissue and blood samples of gastric cancer were collected from the first affiliated hospital of Fujian Medical University following the approval of the hospital’s ethics committee and with the signed consent agreements by patients. Totally, 60 clinically-diagnosed gastric cancer patients, 49 healthy adults participated in this project. Among them, 60 gastric cancer biopsy samples taken from gastroscopy and 51 blood samples were collected. As controls, blood samples were also collected from 49 age-matched healthy adults without gastric abnormalities and free from malignant tumor invasion. Gastric cancerous and the corresponding para-cancerous tissues were dissected during surgery and stored at liquid nitrogen until DNA extraction. The plasma and peripheral blood mononuclear cells (PBMC) were collected from the above patients or healthy adults by centrifuge at 4,000 rpm and stored at –80°C.

The gastric cancer cell lines BGC-823, AGS and human gastric epithelium-immortalized cell line GES-1 or SGC-7901 were obtained from the Shanghai Institute of Digestive Surgery (Shanghai, China) and cultured in an atmosphere of 5% CO_2_ in RPMI-1640 medium supplemented with 10% fetal bovine serum.

### Genomic DNA isolation and bisulfite sequencing analyses

Genomic DNA was extracted by using TaKaRa MiniBEST Universal Genomic DNA Extraction Kit Ver5.0. DNA was bisulfite converted and purified using EpiTect^®^ Plus Bisulfite Conversion Kit (Qiagen, Germany) according to the manufacturer’s instruction.

The above bisulfite-treated DNA was subjected to polymerase chain reaction (PCR). The RPRM primers used for PCR were as follows: 5′-GTTTTAGAAGAGTTTAGTTGTTG-3′ (forward) and 5′-CTACTATTAACCAAAAACAAAC-3′ (reverse). The PCR products were purified by TaKaRa MiniBEST Agarose Gel DNA Extraction Kit (TAKARA, Dalian, China) and then inserted into a pGEM-T easy vector (Promega, WI, USA). Ten recombinant DNA clones were selected from each sample and sequenced (Sangon Biotech, Shanghai, China). The sequencing results were aligned with the RPRM gene reference sequence (NCBI, NC_000002.12) for determining cytosine methylation using the BioEdit software [58], where the cytosines (C) are highlighted in blue in the alignment results (Figure 1).

### Methylation-sensitive melt curve analysis (MS-MCA)

Methylation-sensitive melt curve analysis (MS-MCA) was performed as previously described [40]. Briefly, two control plasmids containing the wild-type and fully C→T converted *RPRM* promoter sequences, designated as SU and SM, were used to generate the unmethylated and fully-methylated melt curve peaks, respectively. The sample DNAs were treated with bisulfite and subjected to real-time PCR by using SYBR^®^ Premix Ex Taq II (Tli RNaseH Plus) (TAKARA, DALIAN, China), and the conditions were as follows: the amplification stage: 95°C 30 s; 95°C 5 s, 56°C 15 s, 72°C 30 sfor 40 cycles; the melt curve stage: 95°C 15 s, 72 to 88°C with 0.1°C increment per cycle. The melt curve was generated and used to determine the methylation status of each sample.

### RNA isolation and semi-quantitative reverse transcription-PCR

The total RNA was extracted from gastric cancer tissue samples and cell lines by Trizol (Thermo Fisher Scientific, USA) according to the manufacturer’s instruction. The reverse transcription reaction was performed using 1 µg of total RNA with PrimeScript^®^ RT reagent Kit plus gDNA Eraser (TAKARA, Dalian, China). The following primers were used for PCR: 5’-CTGGCCCTGGGACAAAGAC-3’ (forward) and 5’-TCAAAACGGTGTCACGGATGT-3’ (reverse) for RPRM; 5’-ACCCACTCCTCCACCTTTG-3’ (forward) and 5’-CTCTTGTGCTCTTGCTGGG-3’ (reverse) for GAPDH. The PCR was conducted using ExTaq HS DNA polymerase (TAKARA, Dalian, China) under the conditions: 94°C denaturation for 5 min, followed by 35 cycles of 94°C 30 s, 55°C 30 s, 72°C 30 s, then 72°C extension for 5 min, finally 4°C to terminate the reaction. The PCR products were separated on a 2% agarose gel.

### Real-time RT-PCR

The mRNA expression levels of DNMT1, DNMT3B and RPRM genes were quantified by the real-time quantitative reverse transcription PCR (qRT-PCR) using SYBR^®^ Premix Ex Taq II (Tli RNaseH Plus) (TAKARA, Dalian, China) at 95°C for 30 s, followed by 40 cycles of 95°C 5 s , 55°C 30 s and 72°C 30 s. The primers used for PCR were as follows: for DNMT1, 5′-GAGGAAGCTGCTAAGGACTAGTTC-3′(forward) and 5′-ACTGCACAATTTGATCACTAAATC-3′(reverse); for DNMT3B, 5′-TACACAGACGTGTCCAACATGGGC-3′ (forward) and 5′-GGATGCCTTCAGGAATCACACCTC-3′ (reverse); for RPRM, 5′-CTGGCCCTGGGACAAAGAC-3′ (forward) and 5′-TCAAAACGGTGTCACGGATGT-3′ (reverse); for GAPDH 5′-CCCTGAGCTGAACGGGAAGCTCAC-3′ (forward) and 5′-CTTGCTGTAGCCAAATTCGTTGCT-3′ (reverse). The PCR was performed on ABI Step One Plus Fast real-time PCR system (Applied Biosystems, USA), and the changes in expression were calculated by using the 2^-△△CT^ method [59].

### Cytotoxicity and demethylation tests of zebularine

AGS or SGC-7901 Cells (5×10^5^ cells per T-75 flask) were treated with the various concentrations (2.5 μM, 12.5 μM, 25 μM, 50 μM, 75 μM and 100 μM) of zebularine for 0, 24, 48, 72 and 96 h. Then, MTT (3-(4,5-dimethyl-2-thiazolyl)-2,5-diphenyl-2-H-tetrazolium bromide) assay was performed as previously described [60]. The absorbance was measured by the microplate reader and the cell viability curves were plotted.

AGS cells were treated with 50 μM and 100 μM of zebularine for 96 h, and SGC-7901 cells with 12.5 μM and 25 μM for 72 h, respectively. After the treatment, the DNAs were extracted and the *RPRM* gene methylation was analyzed by bisulfite sequencing.

### RNA interference, CRISPR/Cas9-mediated gene knockout, and cell transfection

RNA interference was performed by transfection of the shRNA plasmid constructs as previously described [61]. The siRNA sequences used for shRNA construction are following: DNMT1: upstream: 5′-GATCCGCAGGCGGCTCAAAGATTTGCTATGGACACAAATCTTTGAGCCGCCTGCTTTTTTTGTCGACA-3′; downstream: 3′-GCGTCCGCCGAGTTTCTAAACGATACCTGTGTTTAGAAACTCGGCGGACGAAAAAAACAGCTGTTCGA-5′;DNMT3A: upstream: 5′-GATCCGCACTGAAATGGAAAGGGTTTTTCAAGAGAAAACCCTTTCCATTTCAGTGCTTTTTTTGTCGACA-3′; downstream: 5′-AGCTTGTCGACAAAAAAAGCACTGAAATGGAAAGGGTTTTCTCTTGAAAAACCCTTTCCATTTCAGTGCG-3’; DNMT3B: upstream: 5′-GATCCAGGTAGGAAAGTACGTCGCTTCAAGACGGCGACGTACTTTCCTACCTTTTTTTGTCGACA-3′; downstream: 3′-GTCCATCCTTTCATGCAGCGAAGTTCTGCCGCTGCATGAAAGGATGGAAAAAAACAGCTGTTCGA-5′ [62]. The siRNA sequences were inserted into the vector pSilencer2.0-U6-hygro (Ambion, USA) followed by cloning and sequencing verification. The resulting plasmids were introduced to AGS cells by transient transfection (see below).

Transfection was done by using Lipofectamine 3000 Reagent (Thermo Fisher Scientific, USA) according to the manufacturer’s instruction.

DNMTs gene knockout in AGS cell lines was done by using the CRISPR-Cas9 system [63]. The small guide RNA (sgRNA) sequences used for knock out are as follows: DNMT1, 5’GATGTTGCCGAAGAGCCGGT3’; DNMT3A, 5’GAAAGGTACCCGGGTTGTGC3’; DNMT3B, GAAGACTCGATCCTCGTCAA; RPRM, 5’GGGCAGAGGGCGGGTCAGTAThe sgRNA sequences were inserted into the vector PX-459 followed by cloning and sequencing verification. The resulting plasmids were used to generate specific DNMT-null AGS cell clones by transfection and antibiotic selection.

### Western blot analysis

Western blotting was performed as previously described [64]. The cells transfected with the various shRNA constructs were washed with ice-cold PBS, harvested by gentle scraping, and lysed with the protein extraction buffer containing 150 mM NaCl, 10 mM Tris (pH 7.2), 5 mM ethylenediaminetetraacetic acid (EDTA), 0.1% Triton X-100, 5% glycerol, and 2% SDS. The samples were electrophoresed on 16% Tricine-SDS-polyacryomide gels and transferred to polyvinylidene difluoride membranes for hybridization with the corresponding primary antibodies, followed by IRDye 800CW secondary antibody (1:1000) and visualized by Odyssey CLx Western Blot Detection System (Westburg, Netherlands). The expression of GAPDH was used as the endogenous control.

### Confocal microscopy

Confocal microscopy was performed as previously described [65]. The cells were transfected by the corresponding shRNA constructs and the empty vector for 48 hours and then selected in the presence of hygromycin (1 mg/ml) for 5 days. The hygromycin-resistent cells were cultured on the culture chamber slides and used for confocal microscopy analyses. The cells were fixed with 4% paraformaldehyde plus 0.3% Triton X-100 in PBS for 20 minutes. Then the cells were incubated with 5% BSA at room temperature for 30 min. After blocking, the cells were incubated with the rabbit anti-RPRM antibodies (1:200 dilutions) at 37°C for 1h. After washing with PBS for 3 times, each for 10 min, the cells were stained with Alexa Fluor^®^ 488 anti-Rabbit IgG (H+L) secondary antibody (1:1000 dilutions), followed by DAPI staining. The immunofluorescent images were taken using an inverted LSM 780/Axio Imager confocal microscope (ZEISS, Germany). Fluorophores were sequentially excited at 488 nm to prevent cross-excitation. The images were collected and raw data were quantified with ZEN120 Imaging Software.

### Mouse tumorigenesis assay

Female BALB/cAJcl nude mice with 6-week-ages were purchased from Shanghai slack laboratory animal Co. LTD. Mice were subcutaneously injected with SGC-7901 and BGC-823 and their corresponding *reprimo* gene-deficient cell lines on the right shoulders (1 × 10^6^ cells each injection) (one injection per mouse). Each group consisted of 5 mice. The tumor formation was observed 10 days after inoculation.
